# A Simple Mechanism Based on Amino Acid Substitutions is not a Critical Determinant of High Mortality of Japanese Encephalitis Virus Infection in Mice

**DOI:** 10.3390/v10020062

**Published:** 2018-02-03

**Authors:** Yuki Takamatsu, Leo Uchida, Muhareva Raekiansyah, Mark Anthony Luz, Kouichi Morita, Daisuke Hayasaka

**Affiliations:** 1 Department of Virology, Institute of Tropical Medicine, Nagasaki University, 1-12-4 Sakamoto, Nagasaki 852-8523, Japan; yuki.takamatsu@staff.uni-marburg.de (Y.T.); uchidaleo@rakuno.ac.jp (L.U.); m.raekiansyah@gmail.com (M.R.); markanthonyluz@gmail.com (M.A.L.); moritak@nagasaki-u.ac.jp (K.M.); 2 Leading Graduate School Program, Nagasaki University, Nagasaki 852-8523, Japan; 3 Center for Control and Prevention of Infectious Diseases, Nagasaki University, Nagasaki 852-8523, Japan

**Keywords:** Japanese encephalitis virus, pathogenicity, mouse, recombinant, amino acid substitution

## Abstract

For the development of effective treatment strategies for Japanese encephalitis (JE), it is important to identify the viral factors causing severe disease during JE virus (JEV) infection. In this study, we assessed whether amino acid substitutions are critical factors for higher mortality of JaTH160 compared with JaOArS982 in mice using the technique of infectious cDNA clones. We raised the possibility that two amino acids of C_124_ and NS3_482_ of JaTH160 may contribute to increased mortality in mice. However, simultaneous substitutions of these amino acids did not significantly increase the virulence of JaOArS982, suggesting that high mortality due to JaTH160 viral infection cannot be simply attributed to the specific amino acids. Multiple and complex, but not simple, mechanisms may induce the high mortality of JaTH160 infection in mice.

## 1. Introduction

Japanese encephalitis (JE) virus (JEV) causes approximately 30,000 to 50,000 cases and 10,000 to 15,000 deaths in Asian countries annually [1,2]. JEV belongs to the family *Flaviviridae*, genus *Flavivirus* [3,4], whose genomic RNA encodes three structural (C, prM, and E) and seven nonstructural (NS1, NS2A, NS2B, NS3, NS4A, NS4B, and NS5) proteins [5]. The clinical symptoms of JE vary from mild to severe disease, including a non-specific febrile illness, meningitis, encephalitis and meningoencephalitis [6,7]. However, the mechanism of severe central nervous system (CNS) disease has not been fully elucidated.

To evaluate the disease pathogenesis in JEV infection, mice have been employed as a useful infection model [8,9,10]. Mouse models of JEV infection have suggested that several viral and host factors affect disease severity during JEV infection. We previously showed that subcutaneous infection with the JaTH160 strain of JEV causes significantly higher mortality and more pronounced virus propagation in the brains of mice compared with those of the JaOArS982 strain [9]. Thus, we have considered that genome-based comparisons between JaTH160 and JaOArS982 strains would provide useful information for identifying the viral factors responsible for the pathogenicity in JEV infection in vivo.

Previous studies have demonstrated that amino acid substitutions are major factors affecting the virulence in flavivirus infections. For example, it has been shown that amino acid substitutions in the E protein, such as E_49_, E_123_, E_138_, E_176_, E_306_, and E_389/390_ affect the virulence of flaviviruses in the CNS [11,12,13,14], and amino acids, such as C_42,43_, prM_15,17_, NS2A_23_, and NS5_879–881,891_, contribute to the flavivirus pathogenicity in mice [15,16,17,18]. We have also focused on the amino acid substitutions between JaTH160 and JaOArS982 strains to elucidate the pathogenicity in our previous studies.

JaTH160, but not JaOArS982, expresses the NS1’ protein and that NS1’ enhances JEV production in avian cells and embryonated chicken eggs [19]. Previous studies have shown that the NS1’ protein plays a role in the enhanced virulence of the JEV SA14 strain in mice [20]. Thus, we have predicted that NS1’ protein is a critical factor of the higher pathogenicity of JaTH160 compared with JaOArS982. However, our data suggested that NS1’ protein expression in the JaOArS982 strain reduces the mortality in mice, suggesting that the effect of NS1’ on pathogenicity in vivo may vary among virus strains [21].

We also identified a unique amino acid of NS2A_113_ phenylalanine that affects the efficient propagation of JaTH160 in neuroblastoma Neuro-2a cells, but not extraneural origin cells compared with the JaOArS982 strain [22]. Therefore, we predicted that this NS2A_113_ phenylalanine is responsible for the high pathogenicity of JaTH160. However, this amino acid did not affect viral loads in the brain nor survival curves in mice, suggesting that virus propagation in vitro may not reflect the level of virus neurovirulence in vivo [22].

There are 19 amino acid differences between JaTH160 and JaOArS982. Therefore, in the present study, we aimed to identify the amino acids affecting the virulence and to assess whether those amino acid substitutions are critical to determine the high mortality due to JaTH160 infection by a reductionist approach using the infectious cDNA clone techniques of JaTH160 and JaOArS982 strains.

## 2. Materials and Methods

### 2.1. Ethical Statement

The animal experiment protocols were approved by the Animal Care and Use Committee of Nagasaki University (approval number: 091130-2/0912080807 December 8 2009, 100723-1/1008050873 August 5 2010). The animal experiments were performed in accordance with the recommendations in the Fundamental Guidelines for Proper Conduct of Animal Experiment and Related Activities in Academic Research Institutions under the jurisdiction of the Ministry of Education, Culture, Sports, Science, and Technology.

### 2.2. Viruses and Cells

The full-length cDNA clones S982-IC and JaTH-IC were developed from the genome sequences of JaOArS982 and JaTH160 strains, respectively, as described previously [19]. Based on the S982-IC and JaTH-IC, recombinant JEV clones were constructed at the unique restriction enzyme sites, and the point mutations were introduced by site-directed mutagenesis as described previously [19]. The plasmids containing the full-length cDNA clones were linearized and transcribed into RNA using an SP6 transcription kit (mMESSAGE mMACHINE SP6 kit, Life Technologies, Carlsbad, CA, USA) [23]. The RNAs were introduced into baby hamster kidney (BHK) cells by electroporation. Infectious viruses were recovered from those cell culture fluids. Virus stocks were stored at −80 °C until they were used. Viral titers were determined by plaque-forming assays using BHK cells and were expressed as plaque forming unit (PFU)/mL [24]. BHK cells were maintained in Eagle’s Minimal Essential Medium (EMEM) containing 10% fetal calf serum (FCS) and antibiotics.

### 2.3. Mice

C57BL/6j (B6) mice were purchased from Japan SLC, Inc, or Japan CLEA, Inc. Five- to seven-week-old mice were subcutaneously inoculated with 10^4^ PFU of JEV diluted in EMEM containing 2% FCS. Mock infected mice were inoculated with supernatant of cultured BHK cells. Mice were weighed daily, and survivals were recorded for 21 days.

### 2.4. Statistical Analyses

The log-rank (Mantel-Cox) test was used for statistical analysis to assess the significant differences of survival curves between each recombinant virus and S982-infectious clone (IC) virus. A *p* value of <0.05 was considered statistically significant.

## 3. Results

### 3.1. Separate Regions of Viral Genome Affect the High Mortality in Mice

**Figure 1:**
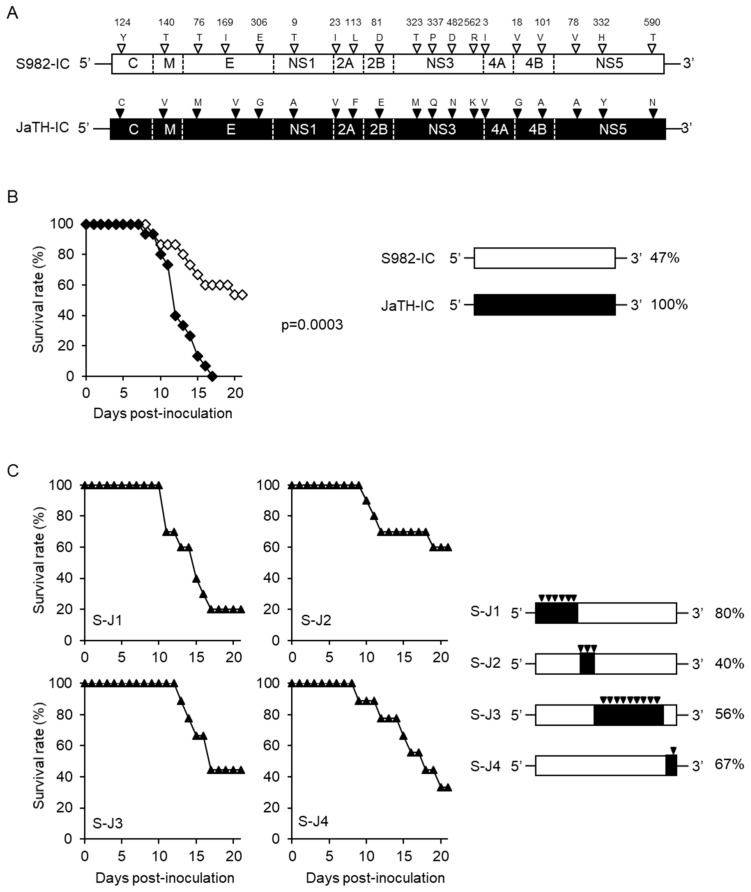
Mortalities of mice infected with recombinant viruses of JaOArS982 and JaTH160 replacing four distinct regions of the viral genome. (**A**) schematic representation of 19 amino acid differences of recombinant S982-IC and JaTH-IC viruses derived from JaOArS982 and JaTH160, respectively. Numbers indicate the position of amino acid in each responsible protein, respectively. (**B**) Survival curves and mortality rates of mice infected with 10^4^ pfu of S982-IC (*n* = 15) and JaTH-IC (*n* = 15) for 21 days. Opened and closed diamonds indicate S982-IC and JaTH-IC, respectively. The log-rank (Mantel-Cox) test was used for statistical analysis to assess significant differences of survival curves. A *p* value of <0.05 was considered statistically significant. (**C**) Survival curves and mortality rates of mice infected with 10^4^ pfu of S-J1 (*n* = 10), S-J2 (*n* = 10), S-J3 (*n* = 9) and S-J4 (*n* = 9) viruses based on S982-IC, in which genome regions 1 (5’ untranslated region (UTR)-NS1_323_), 2 (NS1_324_-NS3_35_), 3 (NS3_36_-NS5_567_), and 4 (NS5_567_-3’UTR) were replaced with those of S982-IC, respectively. Closed triangles indicate recombinant viruses. The black inverted triangles indicate the positions and numbers of amino acids derived from JaTH-IC.

Amino acid differences between JaOArS982 and JaTH160 are shown in Figure 1A. S982-IC and JaTH-IC viruses showed 47 and 100% mortality in mice, respectively (Figure 1B). To address the amino acids affecting the different levels of virulence between these viruses, we applied the reductionist approach, which introduces JaTH-IC viral genome regions into S982-IC, and assessed those recombinant viruses by whether mortality reached high rates, ideally 100%. We expected this approach to narrow down the regions of viral genome stepwise and finally determine the amino acids of JaTH160 responsible for the high mortality in mice.

We first constructed four recombinant viruses (S-J1, S-J2, S-J3 and S-J4) based on S982-IC by introducing four regions of JaTH-IC (Figure 1C). Mortalities of S-J1-, S-J2-, S-J3-, and S-J4-infected mice were 80, 40, 50, and 67%, respectively, and S-J1 virus infection was found to cause higher mortality than others (Figure 1C).

**Figure 2:**
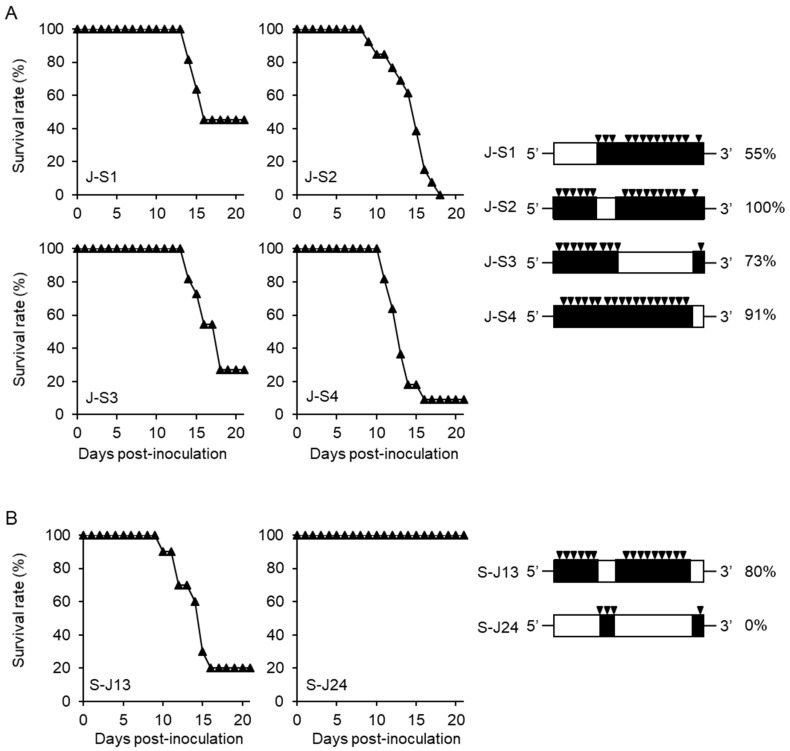
Mortalities of mice infected with recombinant viruses based on S982-IC and JaTH-IC. (**A**) Survival curves and mortality rates of mice infected with 10^4^ pfu of J-S1 (*n* = 11), J-S2 (*n* = 13), J-S3 (*n* = 11), and J-S4 (*n* = 11) viruses based on JaTH-IC, in which genome regions 1, 2, 3, and 4 were replaced with those of S982-IC, respectively. (**B**) Survival curves and mortality rates of mice infected with 10^4^ pfu of S-J13 (*n* = 10) and S-J24 (*n* = 10), whose genome regions 1 and 3, or regions 2 and 4, were of JaTH-IC. Closed triangles indicate recombinant viruses. The black inverted triangles indicate the positions and numbers of amino acids derived from JaTH-IC.

We next constructed recombinant viruses of J-S1, J-S2, J-S3, and J-S4 based on JaTH-IC by replacing the four regions of S982-IC (Figure 2A). Infections with J-S2 and J-S4 viruses caused 100 and 91% mortality in mice, respectively (Figure 2A). On the other hand, infections with J-S1 and J-S3 viruses resulted in 55 and 73% mortality in mice, respectively (Figure 2A).

From these results, we postulated that a combination of distinct regions of 5’UTR-NS1_323_ and NS3_36_-NS5_567_ may contribute to the high mortality due to JaTH-IC virus infection. Thus, we examined the mortality due to S-J13 virus infection in mice, whose regions of 5’UTR-NS1_323_ and NS3_36_-NS5_567_ were of JaTH-IC, and other regions were of S982-IC (Figure 2B). The mortality rate of S-J13 virus-infected mice was 80%, although the survival curve was not significantly different from that of S982-IC virus (Figure 2B).

Although it is controversial as to whether the virulence of S-J13 virus is significantly higher than that of S982-IC virus, we further attempted to focus narrowly on the site of 5’UTR-NS1_323_ and NS3_36_-NS5_567_. Thus, we further constructed recombinant viruses by dividing the 5’UTR-NS1_323_ and NS3_36_-NS5_567_ regions into three and two regions, respectively.

**Figure 3:**
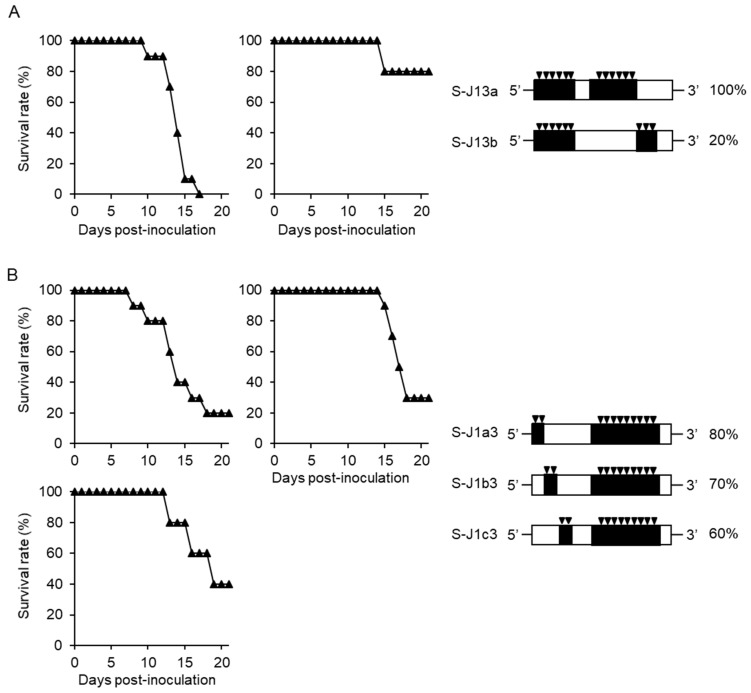
Mortalities of mice infected with S-J13-based recombinant viruses. (**A**) Survival curves and mortality rates of mice infected with 10^4^ pfu of S-J13a (*n* = 10) and S-J13b (*n* = 5) based on S-J13, in which regions 3a (NS3_36_-NS4B_110_) and 3b (NS4B_111_-NS5_567_) were replaced with those of JaTH-IC. (**B**) Survival curves and mortality rates of mice infected with 10^4^ pfu of S-J1a3 (*n* = 10), S-J1b3 (*n* = 10), and S-J1c3 (*n* = 5) based on S-J13, in which regions 1a (5’UTR-M_152_), 1b (M_153_-E_303_), and 1c (E_304_-NS1_323_) were replaced with those of JaTH-IC. Closed triangles indicate recombinant viruses. The black inverted triangles indicate the positions and numbers of amino acids derived from JaTH-IC.

Recombinant viruses of S-J13a and S-J13b were constructed as containing NS3_36_-NS4B_110_ and NS4B_111_-NS5_567_ of JaTH-IC based on S-J1, respectively (Figure 3A). Mortality of S-J13a virus-infected mice was 100%, whereas that of S-J13b infected mice was 20% (Figure 3A). On the other hand, following infections with recombinant viruses of S-J1a3, S-J1b3, and S-J1c3 containing 5’UTR-M_152_, M_153_-E_303_, or E_304_-NS1_323_ of JaTH-IC based on S-J3, respectively, mortalities were 80, 70, and 60%, respectively, and S-J1a3 infection showed a higher mortality (Figure 3B).

From these results, we further focused on the amino acids in the 5’UTR-M_152_ and NS3_36_-NS4B_110_ regions, where viral genome sequences of JaTH160 may induce high mortality in mice.

### 3.2. Amino Acids C_124_ and NS3_482_ of JaTH160 May Affect the High Mortality in Mice

To focus on amino acid substitutions, we next constructed recombinant viruses based on S-J3 and S-J1 by inserting a point mutation that substituted the amino acids in each 5’UTR-M_152_ and NS3_36_-NS4B_110_ region.

**Figure 4:**
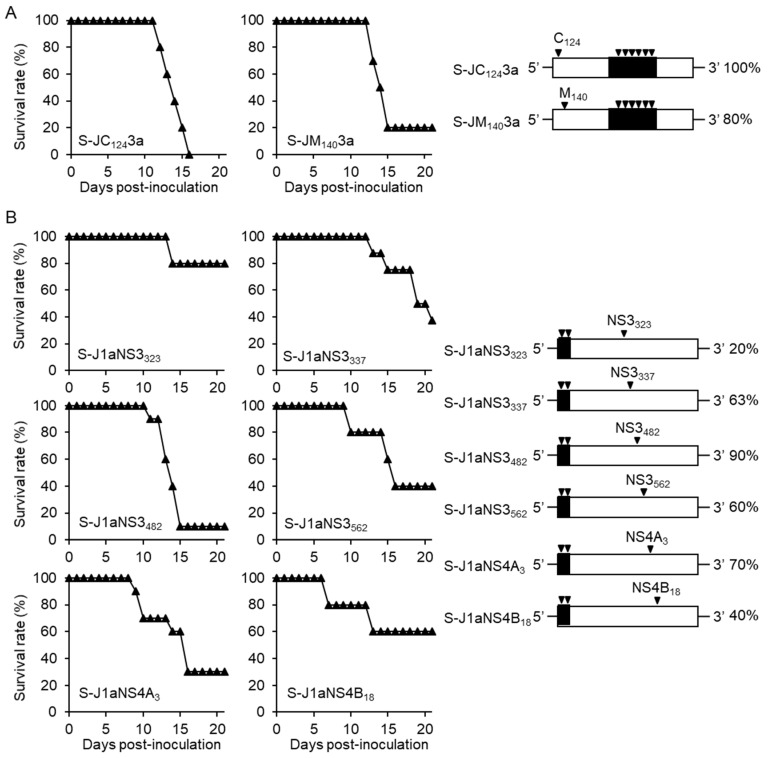

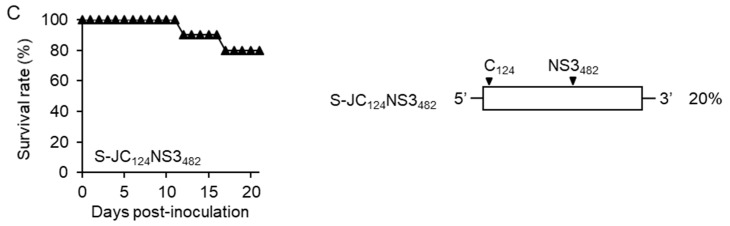
Mortalities of mice infected with recombinant viruses inserting point mutations. (**A**) Survival curves and mortality rates of mice infected with 10^4^ pfu of S-JC_124_3a (*n* = 10) and S-JM_140_3a (*n* = 10) viruses based on S982-IC containing genome region 3a of JaTH-IC, in which amino acids C_124_ and M_140_ were replaced with those of JaTH-IC, respectively. (**B**) Survival curves and mortality rates of mice infected with 10^4^ pfu of S-J1aNS3_323_ (*n* = 5), S-J1aNS3_337_ (*n* = 8), S-J1aNS3_482_ (*n* = 10), S-J1aNS3_562_ (*n* = 5), S-J1aNS4A_3_ (*n* = 10), and S-J1aNS4B_18_ (*n* = 5) viruses based on S982-IC containing genome region 1a of JaTH-IC, in which amino acids NS3_323_, NS3_337_, NS3_482_, NS3_562_, NS4A_3_, and NS4B_18_ were replaced with those of JaTH-IC, respectively. (**C**) Survival curves and mortality rates of mice infected with 10^4^ pfu of S-J C_124_NS3_482_ (*n* = 10) virus based on S982-IC, whose amino acid positions of C_124_ and NS3_482_ were of JaTH160-IC. Closed triangles indicate recombinant viruses. The black inverted triangles indicate the positions and numbers of amino acids derived from JaTH-IC.

There are two amino acid differences in the 5’UTR-M_152_ region between S982-IC and JaTH-IC. We constructed S-JC_124_3a and S-JM_140_3a as containing a point mutation of the position C_124_ and M_140_ based on S-J3, respectively (Figure 4A). After infections with these viruses, mortalities of mice were 100 and 80%, respectively (Figure 4A).

On the other hand, there are six amino acid differences in the NS3_36_-NS4B_110_ region between S982-IC and JaTH-IC (Figure 1A). Thus, we constructed recombinant viruses of S-J1aNS3_323_, S-J1aNS3_337_, S-J1aNS3_482_, S-J1aNS3_562_, S-J1aNS4A_3_, and S-J1aNS4B_18_, to contain each point mutation of the position NS3_323_, NS3_337_, NS3_482_, NS3_562_, NS4A_3_, and NS4B_18_ of JaTH160 based on S-J1a, respectively (Figure 4B). Following infections with these viruses, mortalities of infected mice were 20, 63, 90, 60, 70, and 40%, respectively (Figure 4B).

These results suggested that the amino acids C_124_ and NS3_482_ of JaTH-IC may contribute to the high mortality of JaTH-IC infection, and provide the possibility that a combination of amino acids of C_124_ and NS3_482_ of JaTH160 is crucial for the cause of high mortality in mice.

### 3.3. Simultaneous Substitutions of C_124_ and NS3_482_ are Not Enough to Cause High Mortality

We predicted that simultaneous substitutions of C_124_ and NS3_482_ amino acids into S982-IC can increase mortality in mice. Therefore, to determine whether amino acids C_124_ and NS3_482_ of JaTH160 are responsible for the high mortality in mice, we inserted point mutations as containing C_124_ and NS3_482_ of JaTH160 based on S982-IC (S-JC_124_NS3_482_) and examined the mortality in virus-infected mice (Figure 4C). However, this virus infection resulted in 20% mortality in mice (Figure 4C).

These results indicated that a combination of amino acids C_124_ and NS3_482_ of JaTH160 is not a critical factor of the higher mortality of JaTH160 compared with JaOArS982.

## 4. Discussion

In this study, to assess whether amino acid differences were crucial determinants for the different mortalities between JaTH160 and JaOArS982 infections in vivo, we attempted to specify the amino acids responsible for the high mortality in mice by substituting the amino acids of JaOArS982 to JaTH160. Based on the stepwise approach to narrow down the genome region responsible for the high mortality of JEV using recombinant viruses derived from infectious cDNA clones, we archived two amino acid candidates of C_124_ and NS3_482_ that likely contribute to the increased mortality in mice. However, simultaneous substitutions of these amino acids based on the S982-IC did not result in high mortality like JaTH-IC virus infection in mice. From these observations, we suggest that high mortality due to JaTH160 virus infection cannot be attributed to a simple mechanism derived from specific amino acids of JaTH160, and that multiple and complex mechanisms contribute to the high mortality of JaTH160 virus infection in mice. We propose that a more sophisticated approach is required to elucidate the viral factors that determine the high virulence of JaTH160 in mice.

S-JC_124_3a and S-J1aNS3_482_ viruses induced high mortality in mice. However, the mechanism of the pathogenicity may be different between S-JC_124_3a and S-J1aNS3_482_ viruses. In the case of the S-JC_124_3a virus, the combination of C_124_ and the other amino acids or nucleotide sequences in the region of NS3_36_-NS4B_110_ may be important for its pathogenicity. Moreover, the other amino acids located at close regions or separated regions could influence the C_124_-mediated viral pathogenicity. This theory is also applicable for S-J1aNS3_482_ infection. It is predicted that such substitutions of amino acids and nucleotides may compensate for the function inducing the high mortality of each virus. However, it is likely to be too complex to determine such viral genome factors. A simple reductionist approach is unlikely to be the practical method of obtaining such data.

We previously showed that JaTH160-infected mice developed severe infections of the CNS [9]. On the other hand, JaOArS982-infected mice exhibited varying degrees of encephalitis and different prognoses. We therefore proposed that fatal outcomes are attributable both to immunopathological changes and massive CNS infection [9]. However, it remains unclear whether severe CNS infection of JaTH160 is a direct cause of high mortality in mice or a result of a severe disease course including a specific immune response. In addition, our previous studies of JEV infection have shown that the mortality is not simply determined by neuroinvasiveness, because viral infections in the brains of surviving mice, that did not show apparent clinical signs, were similar levels of viral loads to those of dying mice [9]. These observations raise the possibility that different pathogenicity between JaOArS982 and JaTH160 infections may primarily be attributed to the peripherally induced host responses. Thus, both virus replication itself and host immune response induced by the virus infection should be focused on determining the viral factors for the fatal infection.

We also previously showed the increase of tumor necrosis factor alpha (TNF-α), interferon gamma (IFN-γ), interleukin (IL)-2, and IL-10 in the brain, and the decrease of TNF-α in the spleen in JaTH160 infected-mice [9]. From these observations we suggest that the varied disease symptoms in JE cases are primarily attributed to the immune response in individuals, and secondary viral factors contribute to the induction of such immune responses. Thus, to elucidate the mechanism of severe disease course based on the viral factors is still a priority in the development of effective treatment strategies for JE.
